# Genetic Factors Implicated in the Investigation of Possible Connections between Alzheimer’s Disease and Primary Open Angle Glaucoma

**DOI:** 10.3390/genes14020338

**Published:** 2023-01-28

**Authors:** Grace Kuang, Rebecca Salowe, Joan O’Brien

**Affiliations:** Scheie Eye Institute, Perelman School of Medicine, University of Pennsylvania, Philadelphia, PA 19104, USA

**Keywords:** Alzheimer’s disease, primary open angle glaucoma, dementia, glaucoma, genetic factors

## Abstract

Both Alzheimer’s disease (AD) and primary open angle glaucoma (POAG) are diseases of primary global neurodegeneration with complex pathophysiologies. Throughout the published literature, researchers have highlighted similarities associated with various aspects of both diseases. In light of the increasing number of findings reporting resemblance between the two neurodegenerative processes, scientists have grown interested in possible underlying connections between AD and POAG. In the search for explanations to fundamental mechanisms, a multitude of genes have been studied in each condition, with overlap in the genes of interest between AD and POAG. Greater understanding of genetic factors can drive the research process of identifying relationships and elucidating common pathways of disease. These connections can then be utilized to advance research as well as to generate new clinical applications. Notably, AD and glaucoma are currently diseases with irreversible consequences that often lack effective therapies. An established genetic connection between AD and POAG would serve as the basis for development of gene or pathway targeted strategies relevant to both diseases. Such a clinical application could be of immense benefit to researchers, clinicians, and patients alike. This paper aims to summarize the genetic associations between AD and POAG, describe common underlying mechanisms, discuss potential areas of application, and organize the findings in a review.

## 1. Introduction

Both Alzheimer’s disease (AD) and primary open angle glaucoma (POAG) are diseases of high prevalence and disease burden. AD is the world’s leading cause of cognitive impairment with a worldwide prevalence estimated to be more than 24 million people [1]. Meanwhile, glaucoma is the world’s leading cause of irreversible blindness and approximately 57.5 million people are affected by POAG across the world [2]. Despite emphasized awareness and decades of dedicated research efforts, these two diseases still appear to present more questions than answers. Researchers continue to seek to understand their genetic underpinnings, pathophysiologies, distinct subtypes, and improved therapeutics.

As more research is published in both fields, scientists have taken note of the many similarities reported between these two disorders. Both are diseases of latent onset, progressive global neurodegeneration that leads to debilitating neurologic impairments. They are also now both understood to be heterogeneous collections of diseases with incompletely delineated subtypes [3,4]. Increasing age is the most prominent predisposing risk factor for both diseases, and they share several other risk factors, including ethnicity, history of systemic disease, and family history [1,2,5,6,7].

Beneath these clinical similarities, deeper levels of association have also been previously identified in molecular studies. AD and POAG are postulated to share several commonalities in molecular dysregulation, including disruptions in synaptic function, cellular signaling, axonal transport, and neuronal communication [8]. Other overlapping neurodegenerative pathways include microglia-induced neuroinflammation and excitotoxicity [9]. In recent years, vascular studies have also demonstrated evidence of microvascular dysfunction in both AD and POAG [10]. Moreover, histopathologic and imaging studies have shown similar neuronal degeneration and abnormal protein accumulation patterns in both patient populations, including extracellular abnormal β-amyloid plaques, intracellular phosphorylated tau neurofibrillary tangles, loss of the retinal nerve fiber layer, and reduced brain tissue volumes [11,12,13].

The presence of a considerable number of associations between features at every level of disease is suggestive of related genetics influencing these two conditions. Exploration into shared genetics can supply explanations for the resultant similarities noted in these previously published studies, as well as provide clearer insight into the plethora of unanswered questions across both fields. Knowledge gained in one field could be applied to the other, thereby expediting the research and discovery process. 

Recognition of the integral genetic factors influencing disease pathogenesis could also be instrumental in improving clinical management. Current treatment options for both POAG and AD are relatively limited. Both diseases offer no cure at this time, and available treatments address symptom management and complication prevention. POAG treatment regimens center around lowering intraocular pressure (IOP), which is the major modifiable risk factor for POAG. These include medications such as carbonic anhydrase inhibitors and prostaglandin analogs, and surgical techniques such as trabeculectomies and stent placements [14]. Even in the setting of adequate IOP reduction, approximately 30% of patients experience continued visual decline [15]. Traditional AD therapy only includes pharmacologic agents that aim to improve symptoms of cognitive dysfunction (i.e., acetylcholinesterase inhibitors and an N-methyl-D-aspartate (NMDA) receptor antagonist) [16]. As traditional treatment options operate by preserving the remaining healthy nerve cell integrity, earlier screening based on genetic information could indicate earlier initiation of treatment for the evaluated disease as well as preclinical monitoring for the corresponding disease. In recent years, novel drugs targeting integral processes of disease pathology, such as reversal of β-amyloid plaques, have become approved for clinical application. The current research direction aims to expand treatment targets to involve more etiologies of disease, such as vascular dysfunction and molecular dysregulation [17]. A common goal in AD and POAG therapy research is the development of nerve cell replacement techniques that can replace already lost neuronal function. Greater understanding of genetic contributions to pathophysiology is also crucial for the next step of gene and pathway-based therapies and other strategies with an overarching goal of more effective treatment and improved patient outcomes.

**Figure 1:**
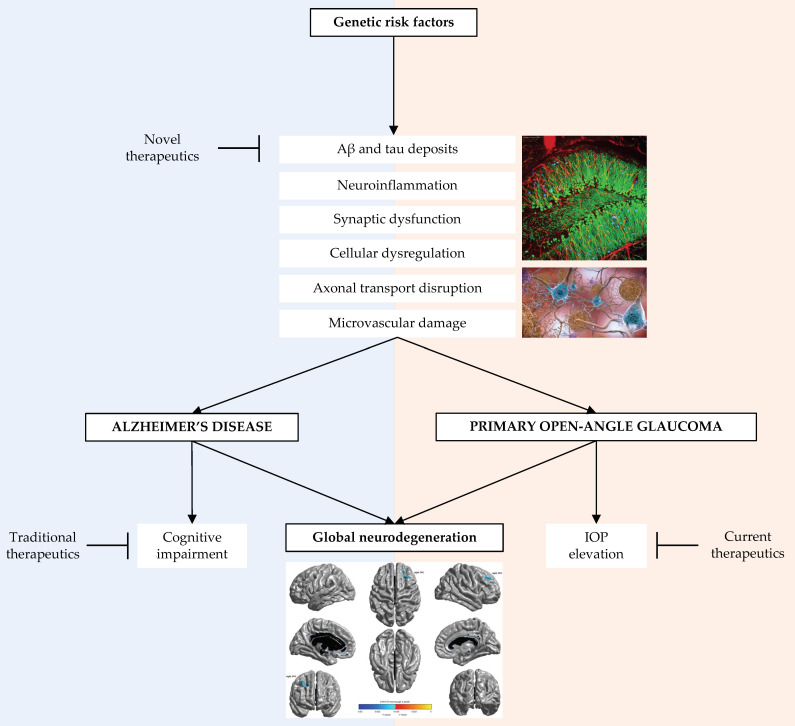
Depiction of the pathologic commonalities between Alzheimer’s disease and primary open-angle glaucoma [18,19,20]. Aβ = amyloid beta; IOP = intraocular pressure.

Overlaps in the common pathologic pathways and targets of therapeutic actions in AD and POAG are portrayed in Figure 1.

Following immense research efforts, 75 risk loci have been associated with AD and 127 risk loci have been associated with POAG [13,21,22]. Researchers utilizing genome-wide association studies (GWAS) data have found multiple protein-coding genes associated with both AD and POAG [21,22]. This review will discuss these previously identified overlapping genes, select additional genes of particular research interest, and the impact that an established genetic connection would have on multiple areas of disease management.

## 2. Overview of Alzheimer’s Disease and Glaucoma

### 2.1. Alzheimer’s Disease

Alzheimer’s disease (AD) is a neurological disease of global cognitive impairment characterized by latent onset, progressive dementia. Dementia refers to a heterogenous syndrome of cognitive decline that is out of proportion to normal biological processes and interferes with functioning [23,24]. Many conditions feature dementia, but AD is the most common cause accounting for 60–70% of all cases [25]. AD risk increases exponentially beginning at 65 years of age, and most cases of AD are late-onset AD (LOAD) that occur after 65 years of age. However, approximately 5% of patients may have early-onset AD (EOAD) prior to 65 years of age [26].

The timeline of AD follows an asymptomatic preclinical phase that can last multiple decades prior to symptom onset, a mild phase marked by limited cognitive impairment, and a final stage of dementia. Although memory decline is the most well-known feature of dementias, patients with AD can present with cognitive decline in a wide range of areas, including memory, behavioral, visuospatial, and language impairments [26]. AD is a diagnosis of exclusion, meaning other known classes of dementia (e.g., vascular dementia, Lewy body dementia, frontotemporal dementia, etc.) must be excluded prior to diagnosis. This rule-out process of AD diagnosis highlights the vague diagnostic criteria and clinical workup of the disease. Diagnosis is difficult to establish as there are currently no definitive diagnostic tests, biomarkers, or imaging studies. Clinical diagnosis can be made utilizing multiple neuropsychological tools, such as cognitive interviews, cerebrospinal fluid (CSF) biomarkers, and positron emission tomography (PET) imaging, but confirmative diagnosis is only possible following postmortem brain examination [27].

Typical features seen in the brains of patients with AD include moderate atrophy of multiple cortical structures, especially the hippocampus and temporal lobes, with associated temporal horn enlargement [28]. Abnormal cleavage of amyloid precursor protein (APP) results in pathogenic β-amyloid protein. Rather than undergoing normal processes of degradation, β-amyloid protein forms aggregates of extracellular deposits called senile plaques that concentrate around cerebral vasculature and in gray matter. This deposition leads to neuronal dysfunction, proinflammatory responses, and neurotoxicity [29]. Increased β-amyloid protein concentrations also induce the formation of intracellular hyperphosphorylated tau neurofibrillary tangles, which is another classic pathologic feature of AD [29].

### 2.2. Glaucoma

Glaucoma is an ophthalmic disease characterized by a loss of retinal ganglion cells (RGC) resulting in optic degenerative neuropathy. Patients with glaucoma typically experience elevated intraocular pressures and progressive loss of peripheral to central vision. Similar to dementias, glaucoma is also a heterogenous collection of diseases. The most common cause of glaucoma is primary-open angle glaucoma (POAG), in which disease pathogenesis cannot be fully explained by an anatomical abnormality, such as in closed-angle glaucoma. Another subset of glaucoma is normal tension glaucoma (NTG), in which symptoms occur in a setting of normal intraocular pressure (IOP) [2].

Patients with glaucoma typically complain of gradual loss of vision that begins peripherally and gradually migrates toward the center of vision. Due to the insidious nature of peripheral visual impairment, most patients do not notice when symptoms first arise. Peripheral fields can be tested on ophthalmic examination, but sensitivity of the exam in early stages of disease is unclear [30]. In cases of POAG with IOP elevations, IOPs can be measured with a Goldmann Applanation Tonometry (GAT) device or other secondary device. The generally accepted normal values of IOP lie in the range of 12 to 21 mmHg, and values outside of this range may indicate need for further evaluation and management. However, it is important to note that IOP is not an absolute diagnostic biomarker for glaucoma. Individuals may suffer from symptomatic NTG in the setting of normal IOP. The opposite can also be true in situations where individuals have multiple recorded elevated IOPs but no symptoms of glaucoma (this condition is termed ocular hypertension) [31]. Another feature heavily utilized in clinical and research settings is cupping or notching of the optic nerve. RGC loss from the surrounding neural rim of the optic nerve leads to an increased or irregular appearance of the center cup that is progressively devoid of neuroretinal tissue, increasing the cup-to-disc ratio.

Traditionally, glaucoma was attributed solely to increased IOP in the ocular system. This force exerts mechanical pressures on the optic nerve head that eventually leads to axonal structural damage and neural cell degeneration [32]. Intraoperative and postoperative imaging studies of ocular structures following open-angle glaucoma surgical treatment have demonstrated that morphological improvements (e.g., Schlemm canal diameter and anterior chamber angle) coincide with IOP reductions and likely improved subsequent outcomes [33,34]. However, IOP elevation is unlikely to be the only etiology, as demonstrated by cases of NTG in which IOP levels are within stable normal ranges. Additionally, disease progression has been found to occur in a significant proportion of patients (10–45%) despite IOP decrease through medical and surgical interventions [13,35]. As a result, multiple other etiologies have been explored in recent years, including primary RGC loss, microglial activation, vascular ischemia, cellular stress, and global neurotoxicity [21,35].

## 3. Background of Connections between Alzheimer’s Disease and Glaucoma

Prior to consideration of similar pathologies, a connection between the eyes and the brain can be identified from the early beginnings of organ development. In approximately the third week of gestation, the forebrain neuroectoderm develops two bilateral indentations in the neural plate that are called optic grooves. As the neural plate closes into the neural tube, these optic grooves continue to extend outwards, growing into optic vesicles anchored to the forebrain by elongated optic stalks. Ultimately, the optic vesicle’s neuroectoderm differentiates into the ocular nervous system, and the corresponding optic stalk becomes the optic nerve that directly connects the brain to the eye [35]. Considering their common embryologic origin, it would be reasonable to expect sustained likeness in the mature organ systems as well as in their disease pathologies.

Indeed, several molecular and histologic similarities have been described in the representative neurodegenerative diseases of each organ system—AD and POAG. Studies in patients with AD who undergo ophthalmological examination have found decreases in the peripapillary retinal nerve fiber layer, RGC loss, and increased optic nerve cup-to-disc ratio [36,37,38,39]. All of these features are classic manifestations of glaucoma. These findings strongly suggest that neurodegeneration in the central nervous system also affects and can be visualized in retinal neurons. Studies in glaucoma have also found that glaucomatous neurodegeneration is not limited to the eye. Several magnetic resonance imaging (MRI) studies in patients with POAG showed evidence of global neurodegeneration throughout the brain with reduced volumes in all structures of the central visual system as well as broader neural structures [12,32,39].

In experimental models, abnormal β-amyloid protein deposition and tauopathy were significantly increased in the lateral geniculate nucleus of rhesus monkey models with glaucoma [40]. Researchers have observed increased caspase-induced abnormal processing of amyloid precursor protein and β-amyloid protein in RGCs of rate models with chronic ocular hypertension [41]. Other common pathways include the insulin receptor pathway thought to be involved in neuronal growth, differentiation, and functioning [42]. Reduced insulin receptor expression is associated with AD, and increased insulin resistance is also associated with IOP elevation [43,44]. Another pathway found to contribute to both disease pathologies involves vascular dysfunction. AD models have demonstrated vascular fragility in capillaries, arterioles, and large arteries such as the Circle of Willis [17]. Vessel damage can result from a variety of factors, including age-related angiogenic decline, decrease in vessel caliber, inefficient cell signaling, and impaired vasodilation [17]. These factors as well as many others ultimately lead to reduced cerebral blood flow, which has been shown to directly correlate with cognitive impairment [45]. Populations of POAG patients have also demonstrated microvascular dysfunction and deficiencies in endothelium-dependent and independent vasodilatory responses [46,47]. Retinal microvascular dysfunction was found in both mild AD and POAG patients as demonstrated by alterations in retinal artery reactivity when compared to healthy controls [10].

Despite numerous studies demonstrating similarities throughout disease presentation, a connection between AD and POAG has yet to be established due to conflicting evidence in the literature. Several population-based studies have demonstrated mixed associations between AD and POAG diagnoses in study cohorts. Some studies have shown that patients with POAG have significantly increased risk of dementia when compared to controls without POAG [48,49,50]. On the other hand, other studies did not identify POAG as a risk factor for AD [51,52,53,54]. 

Of note, a prominent limitation to population-based studies of association between POAG and AD is the reliability of diagnostic information. Most population-based cohort association studies utilize medical records and insurance claims to extract data in study methodology. However, these may not be accurate reflections of true disease prevalence. In a meta-analysis of 23 studies across the world screening for undiagnosed dementia, the overall rate of undetected dementia was approximately 61.7% [55]. In glaucoma population studies, researchers have found that approximately 90% of individuals worldwide and 50% of individuals in developed countries are unaware that they have glaucoma [56,57]. Both AD and POAG are difficult to diagnose due to delayed symptom presentations, variable presentations especially in the early stages of disease, and lack of standardized diagnostic protocols [58]. Another barrier to accurate association studies includes presently undifferentiated subtypes of both diseases. Cohort studies that found no or negative overall associations between POAG and AD found that older age of glaucoma diagnosis and the normal tension glaucoma subtype were associated with dementia [48,51,56,57]. These findings suggest that different subtypes of both AD and POAG may be significantly associated if stratified appropriately.

The literature is currently undecided as to whether a connection exists between AD and POAG. The finding of common genetic connections and molecular pathways could contribute to the establishment of shared connections. Knowledge of genetic contributions could also fortify studies of association through screening for undiagnosed affected individuals, providing genetic tests to aid diagnosis, and clarifying subtypes of these diseases.

## 4. Genetic Factors Relevant to Alzheimer’s Disease and Primary Open Angle Glaucoma

Using publicly available online GWAS datasets in AD and glaucoma, Zheng et al. (2022) identified 49 single nucleotide polymorphisms (SNPs) in 11 risk loci associated with AD and glaucoma (*AGBL2, CELF1, FAM180B, MTCH2, MYBPC3, NDUFS3, PSMC3, PTPMT1, RAPSN, SLC39A13*, and *SPI1)* [22]. In another review using GWAS datasets from 21 studies, Gharahkhani et al. (2021) identified 3 risk loci associated with AD and POAG *(MAPT, CADM2,* and *APP)* [21]. The exact mechanisms of actions for many of these genes remain unclear. Features of currently known gene loci, pathogenic mutations, their molecular pathways, and possible connections to disease pathogenesis based on present literature are described. Previously identified shared genes implicated in the investigation of possible connections between AD and POAG are organized in Table 1.

Many shared gene loci reside within the genomic region of chromosome 11p11.2, including *AGBL2, SPI1, CELF1, FAM180B, MTCH2, MYBPC3, NDUFS3, PSMC3, PTPMT1, RAPSN,* and *SLC39A13*. This chromosome 11 cluster was found to be associated with increased IOP as well as increased risk for POAG [59].

Numerous risk loci involved in a large variety of molecular processes have been identified. Investigation into the pathways that these genes take part in has led to increased recognition of the complexity and multitude of disease etiologies. Some genes of interest have demonstrated integral roles in microtubule structure and function. Microtubules, comprised of repeating tubulin subunits, form tracks within neuronal axons along which molecules necessary for neurocognitive function are shipped during axonal transport [76]. Previous studies in animals and humans have demonstrated that the axonal transport system in AD is defective, resulting in bottleneck accumulation of products within axonal swellings, proteolytic processing of β-amyloid precursor protein, and abnormal hyperphosphorylation of microtubule-associated tau protein [77]. Microtubule disruption has also been mentioned as part of glaucoma pathogenesis, as animal models have demonstrated microtubule deficiency prior to RGC loss [74].

Other genes have been shown to play integral roles in macrophage proliferation and activation via enhanced macrophage colony-stimulating factor (M-CSF) [62]. In AD, deposits of intracellular neurofibrillary tangles and extracellular β-amyloid plaques trigger microglial recruitment for abnormal protein clearance. However, the prolonged proinflammatory effects accompanying this microglial response can lead to neuronal death and exacerbate neurologic dysfunction [61]. Specific variants of pertinent genes resulting in increased expression have been associated with an earlier onset of AD and increased expression of other AD risk genes [63]. Microglia-mediated inflammatory retinal neuron damage has also been seen in experimental glaucoma models, and inhibition of certain aspects of microglial activation has demonstrated neuroprotective effects [9].

The *ε4* allele of the Apolipoprotein E gene (*APOE4*) is considered an established risk factor for AD. *APOE4* has been demonstrated to produce neurons with decreased synaptic function, astrocytes with impaired lipid metabolism, and microglia with reduced β-amyloid phagocytosis [80]. It has also been shown to contribute to the vascular dysfunction etiology through disruptions in the blood–brain barrier, which was previously shown to be associated with early cognitive decline [81,82]. However, research surrounding *APOE4* is inconsistent regarding its impacts on POAG. Several meta-analyses in Asian populations have suggested that *APOE4* leads to similar accelerated neurodegeneration in POAG, whereas other metanalyses concluded that *APOE4* is not associated with glaucoma [42]. A recent study utilizing a large, combined dataset demonstrated that *APOE4* may be protective in POAG through increasing retinal microglial resiliency to neurodegenerative responses [83,84].

## 5. Implications of Genetic Connections between Alzheimer’s Disease and Glaucoma

Presently, patients with AD and glaucoma often suffer from delayed diagnoses and ineffective therapies. Studies in AD populations have shown that patterns of abnormal structural changes and neurodegeneration can occur more than a decade prior to symptom onset [85]. This corresponds with the estimates that 61.7% of patients with dementia and 50–90% patients with glaucoma are undiagnosed [55,56,57]. As a result of the markedly delayed recognition of disease, patients will have undergone years of irreversible neurodegeneration prior to clinic presentation, evaluation, and treatment.

Identification of pertinent genes would introduce the opportunity for asymptomatic screening and counseling in high-risk populations. As currently available therapeutics are only targeted towards symptoms, complications, and attenuating the progression of disease, early diagnosis and treatment are imperative to improve patient outcomes. This would allow for early initiation of currently available therapeutics to preserve residual healthy neurons prior to irreversible neurodegeneration, leading to slower disease progression and better patient outcomes. Genetic testing following symptom presentation of one disease could lead to identification of common pathogenic variants. This would indicate early preclinical screening and monitoring of the other disease before symptom onset. It is important to note that testing and screening of genetic factors may need to take ethnic population and common ancestry into consideration. Various ethnic groups are affected by AD and POAG differently due to anatomic characteristics, systemic conditions, and genetic predispositions. For example, in subgroup analyses of a large meta-analysis, *APOE4* was significantly associated with risk of POAG in Asians but not in Caucasians [86]. In both POAG and AD, African Americans have been found to have a higher prevalence, more rapid progression of disease, and greater severity of disease as compared to their white peers [5,6]. Increased study focused on specific ethnic populations and their relation to the disease processes can help to further elucidate the impact of genetic contributions and potential for screenings, while ensuring the inclusion of the most affected populations in these future steps.

Furthermore, knowledge of the genetic underpinnings and contributory molecular pathways for disease pathogenesis would be the first step towards novel gene or pathway-based therapies. Presently employed treatment strategies only target disease symptoms, progression, and complications [25,87]. These include cholinesterase inhibitors (e.g., donepezil, etc.) and glutamate regulators (e.g., memantine, etc.). In glaucoma, a variety of medications targeting different pathways of IOP management have been employed. These include beta-adrenergic antagonists (e.g., timolol, etc.), alpha-adrenergic agonists (e.g., brimonidine, etc.), cholinergic agonists (e.g., pilocarpine, etc.), carbonic anhydrase inhibitors (e.g., dorzolamide, etc.), prostaglandins (e.g., latanoprost, etc.), and others. Surgeries to lower intraocular pressure are also used.

There are currently no cures; thus, AD and glaucoma remain as irreversible diseases. The scientific community has long recognized the need for effective treatments that act upon the pathologic mechanisms of disease, and recent pharmacologic developments have been directed towards disease modifying therapeutics (DMT). In June 2021, the U.S. Food and Drug Administration (FDA) granted a landmark approval of the first drug to target the underlying pathologies of AD: aducanumab. Aducanumab is a monoclonal antibody that selectively targets and clears pathogenic β-amyloid plaques. As more momentum is gained towards the development of DMTs, a step further from anti-pathologic therapies would be genetic or pathway specific therapies that directly impact pathogenesis at the molecular level rather than downstream presentations. While still in the early stages of development, gene therapies for AD and POAG have proven promising in animal studies, and clinical trials are currently ongoing [88,89]. Understanding genes with common roles between AD and POAG could accelerate this process of novel diagnostic and therapeutic applications.

## 6. Conclusions

Underlying connections between Alzheimer’s disease and glaucoma remain to be firmly established, but multiple common genetic undercurrents identified between the two disease entities suggest related molecular pathways and pathophysiologies. Further studies are necessary to elucidate the precise mechanisms by which shared genetics manifest as disease similarities visualized in previous clinical studies. Greater understanding of the genetic associations between AD and POAG would drive collaboration and exchange of knowledge between these two fields of study as well as having potential application to understanding other forms of neurodegeneration. Furthermore, new points of entry into future developments of disease screening, management, and treatment could potentially be introduced. Interplay of knowledge between genetic research and clinical studies, AD and glaucoma, as well as current symptomatic strategies and novel disease modifying diagnostics and therapeutics is expected to yield findings beneficial to the scientific and general medical communities.

## Figures and Tables

**Table 1:** Shared genes in studies of connection between Alzheimer’s disease and primary open-angle glaucoma [8,9,21,22,40,41,59,60,61,62,63,64,65,66,67,68,69,70,71,72,73,74,75,76,77,78,79].

Gene	Name	Location	Protein Product	Function	Pathogenic Mutation	Association with AD	Association with POAG
*AGBL2*	ATP/GTP-binding protein-like 2	11p11.2	Cytosolic carboxypeptidase 2 (CCP2) enzyme	Catalyzes post-translational modification of ɑ-tubulin subunit	rs11604825 rs11602395	Microtubular axonal transport alteration	IOP elevation
*MAPT*	Microtubule associated protein tau	17q21.31	Microtubule-associated protein tau (MAPT)	Encodes tau proteins and other mRNA products	Post-translational alternative splicing and modification	Tau structure isoform variations	Misbalance in glaucomatous RGC axons
*SPI1*	Spi-1 proto-oncogene	11p11.2	PU.1 erythroblast transformation specific (ETS) transcription factor	Promotes gene expression of hematopoietic cell lineages	28 SNP’s	Microglia activation	Microglia activation, IOP elevation
*CELF1*	CUG triplet repeat, RNA binding protein 1 (CUGBP) embryonic lethal abnormal vision (Elav)-like family member 1	11p11.2	CELF/BRUNOL protein	Regulates pre-mRNA splicing and mRNA expression	rs4752845 rs34958982 rs66749409 rs12798346 rs56400411	Alternative splicing of tau protein	IOP elevation
*FAM180B*	Family with sequence similarity 180 member B	11p11.2	Family with sequence similarity 180 member B	Presently unknown function	rs11605348	Expressed at higher levels in brain	IOP elevation
*MTCH2*	Mitochondrial carrier 2	11p11.2	Solute carrierfamily 25 (SLC25) family nuclear-encoded mitochondrial transporters	Involved in apoptotic pathway via recruitment of Bcl-2 family BID protein	rs4752856 rs4752856 rs4752856	Disrupts mitochondrial motility, metabolism, and function	IOP elevation
*MYBPC3*	Myosin binding protein C3	11p11.2	Cardiac isoform of myosin binding protein C (MyBP-C)	Regulates cardiac striated muscle contraction	rs2856661	May affect axonal growth and synaptic development	IOP elevation
*NDUFS3*	NADH:ubiquinone oxidoreductase core subunit S3	11p11.2	Iron-sulfur protein (IP) component in mitochondrial NADH:ubiquinone oxidoreductase (complex I)	Involved in mitochondrial electron transport chain and cellular functions	rs2030166 rs2030166	Increased levels in early stages of mild cognitive impairment	IOP elevation
*PSMC3*	Proteasome 26S subunit, ATPase 3	11p11.2	26S proteasome	Involved in ubiquitin-proteasome degradation system	rs11600581	Increased cellular proteotoxic stress	IOP elevation
*PTPMT1*	Protein tyrosine phosphatase mitochondrial 1	11p11.2	Phosphatidylglycerophosphatase and protein-tyrosine phosphatase 1	Involved in mitochondrial metabolic pathways and embryogenesis	rs56400411 rs7945473	Mitochondrial dysfunction	IOP elevation
*RAPSN*	Receptor associated protein of the synapse	11p11.2	Receptor-associated protein of the synapse	Anchors postsynaptic nicotinic acetylcholine receptors	rs35705029	Acetylcholine neurotransmitter signal alteration	IOP elevation
*SLC39A13*	Solute carrier family 39 member 13	11p11.2	Zinc transporter transmembrane protein	Facilitates zinc transport across membranes	rs755554	Disturbance of zinc homeostasis	IOP elevation
*CADM2*	Cell adhesion molecule 2	3p12.1	Synaptic cell adhesion molecule 1 (SynCAM)	Interacts with cytoskeletal proteins for cellular support and structure	r71316816 rs13101042 rs2220243	Impaired synaptic adhesion and maintenance	Expressed almost exclusively in brain and retina and found to have altered expression throughout all stages of glaucoma
*APP*	Amyloid beta precursor protein	21q21.3	Amyloid precursor protein (APP)	Acts as a transmembrane cell surface receptor and regulates neuronal functions	rs59892895	Abnormal cleavage forms β-amyloid plaques	Increased abnormal expression in glaucoma models
*APOE*	Apolipoprotein E	19q13.32	Apolipoprotein E	Maintains lipid metabolism and balance in circulatory and peripheral systems	*ε4* allele	Increased risk for disease, increased amount of β-amyloid plaques	Reduced risk for disease, decrease activation of microglia

AD = Alzheimer’s disease; POAG = primary open angle glaucoma; IOP = intraocular pressure; SNP = single nucleotide polymorphism; ATP = adenosine triphosphate; GTP = guanosine triphosphate; Bcl-2 = B-cell lymphoma 2; BID = BH3 interacting-domain death agonist; NADH = nicotinamide adenine dinucleotide.

## Data Availability

No new data were created or analyzed in this study. Data sharing is not applicable to this article.
